# Anaerobic Codigestion of Sludge: Addition of Butcher’s Fat Waste as a Cosubstrate for Increasing Biogas Production

**DOI:** 10.1371/journal.pone.0153139

**Published:** 2016-04-12

**Authors:** E. J. Martínez, M. V. Gil, C. Fernandez, J. G. Rosas, X. Gómez

**Affiliations:** 1 Chemical and Environmental Bioprocess Engineering Group, Natural Resources Institute (IRENA), University of León, León, León, Spain; 2 Instituto Nacional del Carbón, INCAR-CSIC, Oviedo, Asturias, Spain; Duke University Marine Laboratory, UNITED STATES

## Abstract

Fat waste discarded from butcheries was used as a cosubstrate in the anaerobic codigestion of sewage sludge (SS). The process was evaluated under mesophilic and thermophilic conditions. The codigestion was successfully attained despite some inhibitory stages initially present that had their origin in the accumulation of volatile fatty acids (VFA) and adsorption of long-chain fatty acids (LCFA). The addition of a fat waste improved digestion stability and increased biogas yields thanks to the higher organic loading rate (OLR) applied to the reactors. However, thermophilic digestion was characterized by an effluent of poor quality and high VFA content. Results from spectroscopic analysis suggested the adsorption of lipid components onto the anaerobic biomass, thus disturbing the complete degradation of substrate during the treatment. The formation of fatty aggregates in the thermophilic reactor prevented process failure by avoiding the exposure of biomass to the toxic effect of high LCFA concentrations.

## Introduction

Environmental concerns and stringent regulations regarding sludge management and disposal have made it necessary to seek alternatives and feasible solutions for the treatment of such waste. On the other hand, food-processing industries generate wastewaters and solid fatty by-products, which are difficult to treat and can cause environmental problems when improperly disposed. The main treatment option is incineration but this, results in increased management costs. Lipid-rich materials, which are abundant in food-processing industries, are known to have high biogas potential. This characteristic makes them an interesting cosubstrate for treatment in anaerobic reactors [1, 2]. In the case of butcheries, due to changes in customer perception of a healthy diet, the amount of animal fat discarded suitable for human consumption is increasing. The excessive consumption of fats has been associated with higher rates of obesity and increased risk of chronic diseases [3]. In addition, the meat industry also needs to deal with the negative perception caused by the high carbon footprint of the business [4, 5]. Therefore, the valorization of butcher’s animal fat for biogas production will aid in increasing green credentials of the meat industry by means of producing a renewable fuel and allowing the recycling of nutrients.

Anaerobic digestion is a well-established process for converting organic matter into biogas and digestate, with the latter being a valuable fertilizer and soil conditioner. In the past two decades, anaerobic digestion has been applied as an effective technology for solving the energy shortage [6]. Codigestion offers several benefits over digestion of separate materials. Codigestion of sewage sludge with other organic wastes improves biogas production and organic matter degradation [7], and it also involves the dilution of inhibitory compounds and favors the degradation of lipids [8].

Despite the reported benefits of codigestion, the accumulation of lipid components during the digestion process may cause inhibition of the process, associated with the toxicity of a given number of fatty acids on anaerobic microorganisms. The main long-chain fatty acids (LCFAs) found in food-processing industries are oleic acid (C18:1), which is mainly found in olive, pecan, and teased oils, and stearic acid (C18:0), which is present in cocoa, tallow, and lards [9]. Under anaerobic conditions, these LCFAs are produced when fats and oils are hydrolyzed. Several studies have suggested that inhibition caused by these acids could be reversible, with acclimatization being a key factor in avoiding negative effects on microbial communities [1, 10, 11].

Long-chain fatty acids inhibit H_2_-producing bacteria responsible for β-oxidation [12, 13] and may disrupt the process in different ways. Adsorption of LCFAs onto the sludge can affect the transport and protective functions of the bacterial wall and form a hydrophobic layer of LCFAs around biomass aggregates, thus reducing the mass transfer between the media and bacteria [10]. Entrapment of LCFAs in biomass aggregates can lead to biomass flotation inside the reactor and, as a consequence, to biomass washout [14, 15]. Precipitation of LCFAs with divalent ions such as Ca^2+^ or Mg^2+^ makes them inaccessible to the anaerobic biomass and hence reduces their biodegradability [11], but may also aid in avoiding biomass toxicity.

Many experimental approaches have been used to study interactions of LCFAs in anaerobic digestion of lipid-rich materials, including potential toxicity, microbial communities, mathematical modeling, and microscopic and macroscopic analyses [16, 17]. Fourier-transform infrared spectroscopy (FTIR) is a spectroscopic tool that provides valuable information regarding the chemical characteristics of samples. This technique is sensitive to changes in organic functional groups, and it has been demonstrated to be well suited for the study of organic transformations during biological processes [18–20].

Another important parameter is the temperature during the digestion process. Mesophilic conditions are commonly studied at 36–40°C, while thermophilic conditions are usually tested at around 55°C. An increase in the temperature up to the thermophilic regimen would allow for an increase in the treatment capacity of the digester due to the capability of thermophilic microorganisms to work at higher organic loading rates (OLRs) and lower hydraulic retention times (HRTs).

The aim of the present research was to assess the effect of animal fat obtained from butcher’s shops when added as a cosubstrate on the anaerobic digestion of sewage sludge. Anaerobic codigestion with animal fat was conducted using mesophilic and thermophilic regimens to evaluate the combined effect of temperature and high accumulation of LCFAs. The characteristics of the digested material were evaluated by the use of FTIR spectroscopy.

## Material and Methods

### Characteristics of substrate and inoculum

Sewage sludge (SS) and inoculum were obtained from the wastewater treatment plant of Cáceres (Spain). The plant has an anaerobic digester for the treatment of a mixture of primary and secondary sludge, and operates under a mesophilic regimen. The secondary sludge was obtained from the activated sludge process using a high biomass retention time, resulting in aged aerobic sludge. Animal fat (F) was collected from a local butcher’s shop (León, Spain). The material was comprised of animal fat discarded by clients due to excessive fat content of veal meat. The chemical characteristics of the materials used in this study are presented in Table 1.

**Table 1:** Characterisation of substrates.

Parameter	Inoculum	SS	F
**TS (g L**^**-1**^**)**	12.3±0.6	19.0±0.9	98.3±4.9
**VS (g L**^**-1**^**)**	8.0±0.4	14.9±0.7	98.2±4.9
**Alkalinity (mg L**^**-1**^**)**	1316±65	512±26	-
**NH**_**4**_^**+**^ **(mg L**^**-1**^**)**	276±14	137±7.0	-
**Organic Matter (%)**	0.87±0.04	1.17±0.05	98.1±5.0
**Kjeldahl Nitrogen (%)**	0.1	0.1	0.04
**C/N**	4.7	6.6	1438.7
**Lipids (%)**	n.d	n.d	0.27±0.01
**LCFAs (%)**			
**Myristic C:18**	n.d	n.d	4.43±0.22
**Palmitic C:16**	n.d	n.d	21.35±1.25
**Stearic C:18**	n.d	n.d	73.53±4.53

(n.d) not detected, TS: Total solids; VS: Volatile solids.

### Experimental set-up

#### Batch digestion

The biochemical methane potential (BMP) of substrates was determined under batch conditions. Experiments were carried out in Erlenmeyer flasks of 250 mL. The temperature was controlled by a water bath and set at 35 ± 1°C for mesophilic and 55 ± 1°C for thermophilic tests. Agitation was provided by means of magnetic stirrers. Bottle gasometers were used to measure the volume of gas. Measurements were standardized to temperature and pressure (STP) of 0°C and 760 mmHg, respectively. Erlenmeyer flasks were inoculated using a ratio of VS inoculum to substrate (I:S) in the range of 2:1 to 1:1. This ratio was selected to avoid the addition of alkali solution for pH control. Batch digestion systems were denoted as MS when digesting sewage sludge under mesophilic conditions and TS under thermophilic conditions. In a similar way, codigestion systems were denoted as MS:F and TS:F. These systems were evaluated by adding fat at percentages of 2.5% and 5% w/v.

#### Semi-continuous digestion

Semi-continuous digestion was carried out in completely stirred reactors. The working volume was 3 L for the mesophilic reactor and 5 L for the thermophilic reactor. Based on batch results, the start-up of thermophilic reactors was carried out by applying a 40-day acclimation period with low organic loading rates using sewage sludge as the sole substrate. Reactors were equipped with mechanical agitators and outer-jackets to circulate heating water. Temperature was controlled at 35 ± 1°C and 55 ± 1°C in accordance with the digestion regimen. Reactors also had sampling ports for the withdrawal of liquid samples and gas collection. Feeding was manually performed once a day. Samples were taken after thoroughly mixing and prior to feeding. Reactors were evaluated at an HRT of 30 days, except for the reactor digesting sewage sludge at mesophilic conditions denoted as RMS, which was tested at an HRT of 40 days since the application of lower digestion times resulted in acidic conditions. Semi-continuous reactors were denoted as RTS when digesting sewage sludge under thermophilic conditions, while semi-continuous codigestion reactors were denoted as RMS:F when codigesting sewage sludge and fat under mesophilic conditions and RTS:F when codigestion was performed under thermophilic conditions.

#### Analytical techniques

Total and volatile solids (TS, VS), pH, ammonia, and alkalinity were measured in accordance with APHA Standard Methods [21]. Nitrogen concentration was measured by the Kjeldahl method. Organic matter was analyzed in accordance with the Walkley–Black method [22]. Lipid content was measured by Soxhlet extraction using Velp Scientifica SER 148/3 extractor in accordance with APHA Standard Methods [21].

Biogas composition was analyzed as described in Martínez et al. [23] using a gas chromatograph (Varian CP3800 GC) equipped with a thermal conductivity detector. A packed column (HayeSep Q 80/100; 4 m) followed by a molecular-sieve column (1 m) was used to separate CH_4_, CO_2_, N_2_, H_2_, and O_2_. The carrier gas was helium, and the columns were operated at a pressure of 331 kPa and a temperature of 50°C.

Volatile fatty acids (VFAs) were measured as described by Cuetos et al. [24] using a gas chromatograph and a flame ionization detector (FID) equipped with a Nukol capillary column (30 m × 0.25 mm × 0.25 μm) from Supelco. The carrier gas was helium. The injector and detector temperatures were 220°C and 250°C, respectively. The oven temperature was set to 150°C for 3 min and increased to 180°C with a ramp of 10°C min^-1^. The detection limit for VFA analysis was 5.0 mg L^-1^. The system was calibrated with a mixture of standard volatile acids from Supelco (for the analysis of fatty acids C2 to C7). Samples were previously centrifuged (10 min, 3500 × g) and the supernatant was filtered through 0.45-μm cellulose filters.

Gas chromatography was used for the analysis of LCFAs. Samples were obtained at the end of the semi-continuous experiments after dismantling of reactors. Samples were extracted with n-heptane. The solution was then centrifuged for 30 min at 3500 × g and filtered through a 0.2-μm Millipore Millex-FGS filter. The sample was injected into a Perkin-Elmer AutoSystem XL chromatograph equipped with an FID detector and a PEG (100% polyethylene glycol) column (15 m × 0.53 mm × 0.5 μm). The carrier gas was helium. The initial oven temperature of 100°C was maintained for 1 min and then increased to 250°C with a ramp of 5°C min^-1^; this temperature was maintained for 5 min. The injector and detector temperatures were 250°C and 275°C, respectively. The system was calibrated using a mixture of LCFAs from individual acids with concentrations in the range of 0 to 100 mg L^-1^. The detection limit for LCFA analysis was 5.0 mg L^-1^. The acids analyzed were C8 to C18 (with even numbers of carbon atoms) and all were obtained from Sigma.

### FTIR analysis

Samples were dried at 105°C in a furnace for 48 h, and then ground in a laboratory ball mill (Retsch mill model MM200) before analysis. Two milligrams of each dried milled sample was ground with 200 mg KBr (FTIR grade) and homogenized. KBr pellets were compressed under vacuum in a standard device under pressure of 6000 kg cm^-1^ for 10 min. Infrared spectra were recorded using an FTIR Perkin-Elmer 2000 spectrophotometer over the 4000 to 400 cm^-1^ range at a rate of 0.5 cm s^-1^. Fifty scans were collected, averaged for each spectrum, and corrected against ambient air as background, as described by Cuetos et al. [24]. Digestate samples from semi-continuous experiments and SS feedstock were analyzed. Spectra are represented as the mean values of three replicates for each sample. Digestate samples were obtained at the end of experiments prior to dismantling the semi-continuous reactors. Sewage sludge and commercial LCFAs (palmitic and stearic acids) were also analyzed to facilitate the interpretation of the spectra. Moreover, spectra were vector-normalized for comparison following the procedure proposed by Meissl et al. [25].

#### Statistical analysis of FTIR spectra

Two multivariate statistical methods, Hierarchical Cluster Analysis (HCA) and Principal Component Analysis (PCA), were used for the evaluation of the samples based on their FTIR spectra witin the region 4000–800 cm^-1^. The initial data values were standardized mean centered, and auto-scaled to variance 1 prior to analysis to avoid any effects of scale of units that they were measured with.

HCA was used to classify the studied samples into different groups, based on the values of the set of data extracted from the FTIR spectra. The clusters were formed by grouping samples according to their similarity, and the results are presented in the form of dendrograms. Grouping in clusters was carried out using Ward’s algorithm.

PCA was used in order to reduce the initial data from linear combinations of the original variables. The aim of this technique is the reduction of an original set of variables into a smaller set of non-correlated components that represent most of the information in the original variables. Usually, only the first few principal components in a descending order explain the maximum of the total variance of all original variables. The score plot of the first principal components was used to investigate the inter-relationships between the samples, as it allowed the observation of clusters of samples. Both HCA and PCA were performed using SPSS v. 19.0 software.

## Results and Discussion

### Batch digestion

**Fig 1:**
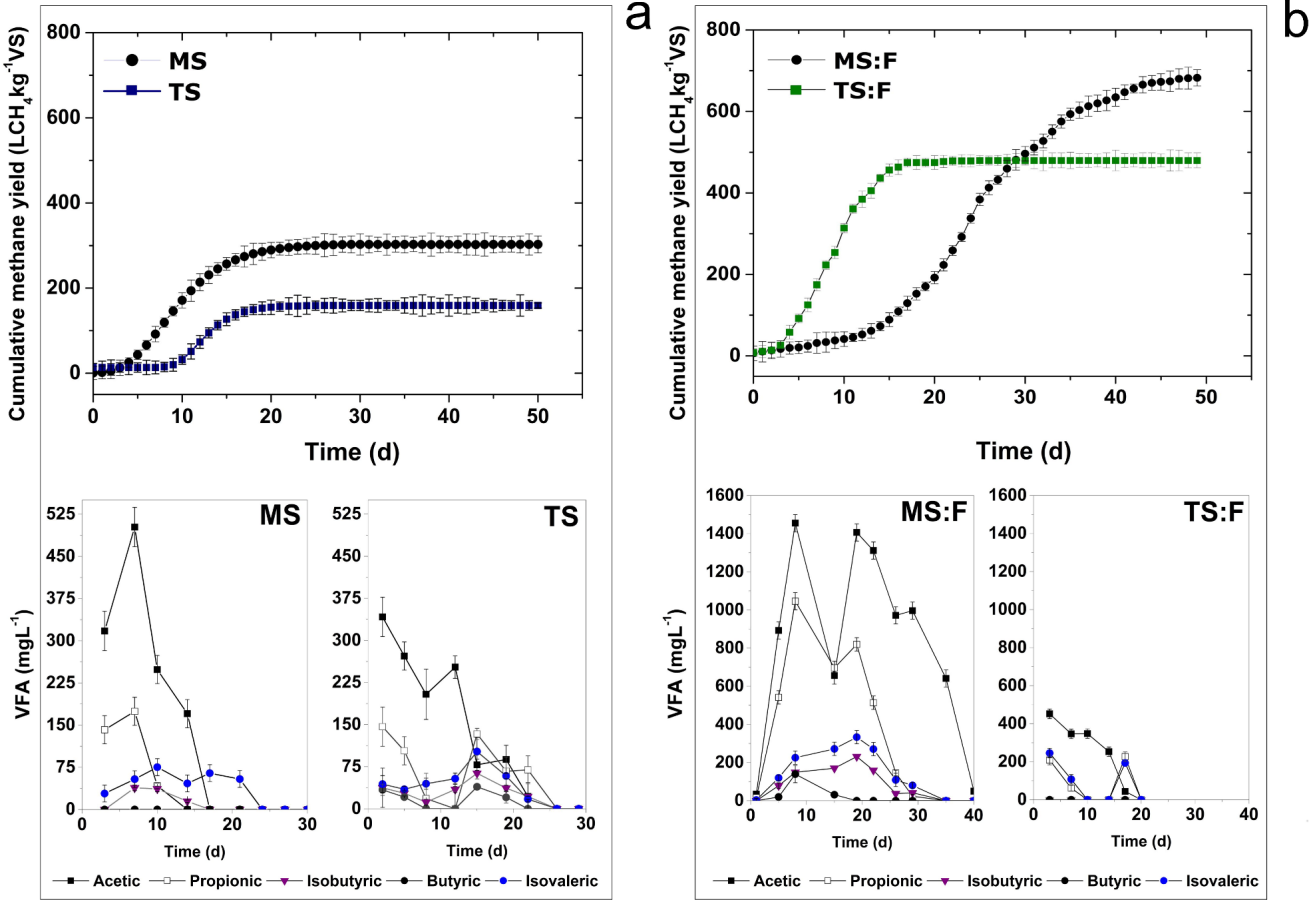
**Batch digestion experiments of sludge:** (a) methane yield and VFA results of mesophilic (MS) and thermophilic (TS) and (b) co-digestion experiments of sludge with fat: methane yield and VFA results of mesophilic (MS:F) and thermophilic conditions (TS:F).

Results of methane yield and VFAs obtained from batch digestion experiments of sludge and codigestion with animal fat at mesophilic and thermophilic conditions are presented in Fig 1A and 1B. The digestion of sludge was initially performed under mesophilic conditions at an I:S ratio of 1. However, the rapid acidification of sludge prevented further digestion of the substrate (data not shown). A second experiment was then performed with an I:S ratio of 2. Results are shown in Fig 1A where an initial accumulation of VFAs was observed followed by subsequent degradation.

In contrast, the thermophilic digestion was carried out using an I:S ratio of 1. Once the lag-phase had been overcome, the conversion of the organic material was achieved. The final value obtained for the specific methane production was, however, much lower than that under mesophilic conditions. The thermophilic experiment was performed using a mesophilic inoculum; thereby, an extended lag-phase was expected in this experiment associated with the acclimation to higher temperatures. The poor results may be explained by the prolonged inhibitory state experienced by this reactor, and by the fact that the experiment was performed using a non-adapted inoculum to thermophilic conditions. Furthermore, the thermophilic system did not show high VFA accumulation. This behavior could be explained by the lower capacity of the non-adapted anaerobic microflora to degrade the organic material. VFA produced during the acidogenesis phase accumulated in the system and probably caused an inhibitory stage of acetoclastic methanogenic activity. This unbalanced reaction rate between acidogens and methanogens implies that solubilized compounds (i.e., VFAs) at the thermophilic temperature were not efficiently converted to CH_4_ [26].

Results from batch codigestion tests are presented in Fig 1B. There was a significant increase in biogas production under the mesophilic regimen when fat was used as a cosubstrate. The benefits of codigestion when adding this cosubstrate are due to the increase attained in OLR and thus in the effective use of the reactor volume, rather than to an increase in the specific methane production. During the initial stage of digestion, the accumulation of VFAs, along with a lag-phase in the cumulative gas curves, was observed. Under thermophilic conditions, the system was capable of rapidly degrading complex lipid materials that were added with the cosubstrate, and thus VFA build-up was not a cause of inhibition. The gas yield obtained during this experiment was lower than that of its homologous mesophilic reactor, even though an extended adaptation period to thermophilic conditions was applied to the inoculum.

### Semi-continuous digestion

**Fig 2:**
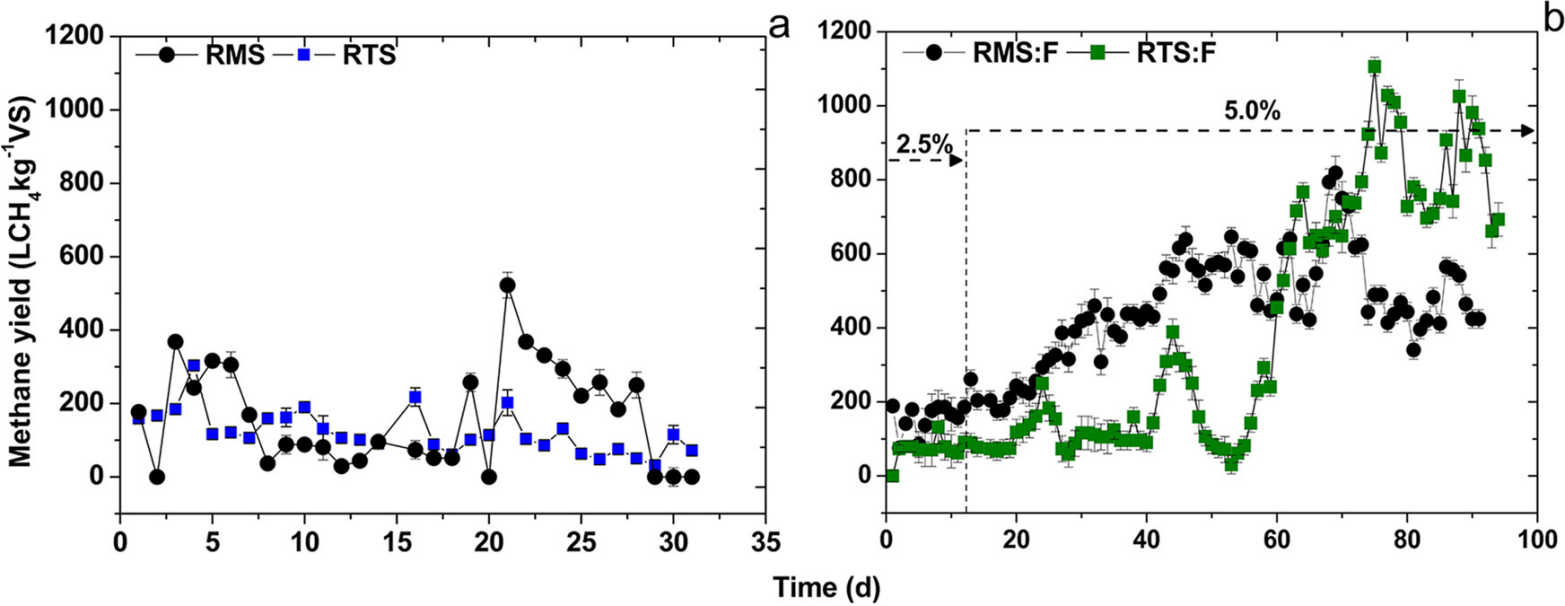
**Methane yield obtained from digestion:** (a) sludge under mesophilic (RMS) and thermophilic (RTS) conditions and (b) co-digestion with fat under mesophilic (RMS:F) and thermophilic (RTS:F) regimens. Percentage (w/v) in figure indicates the amount of fat added as co-substrate.

Methane yields obtained from RMS and RTS reactors are presented in Fig 2A. Low gas production was observed over the entire test with periods of nil gas production under mesophilic conditions. Digestion of this particular sludge proved to be difficult with the need for repetitive inoculation steps to increase the pH of this system. Low alkalinity of the reactor resulted in recurrent pH variations being observed during the experiment. It was finally possible to achieve a decrease in VFA content (average value of 529 mg L^-1^) during the start-up phase to the low values reported in Table 2, but at the cost of operating at low OLRs (0.25 g VS L^-1^ d^-1^ equivalent to an HRT of 40 d).

**Table 2:** Parameters of semi-continuous digestion at mesophilic and thermophilic conditions.

Parameter	MS	TS	MS:F			TS:F		
**% Co-substrate**	0	0	2.5	5	5	2.5	5	5
**Days of experiment**	32	32	12	17	58	12	18	60
**OLR (gVS L**^**-1**^ **d**^**-1**^**)**	0.2±0.1	0.3±0.1	0.94±0.2	1.3±0.2	1.2±0.2	0.8±0.2	1.3±0.2	1.1 ±0.2
**Biogas (mL d**^**-1**^**)**	284±30	500±50	444±42	1167±60	1894±80	669±60	1460±80	4560±20
**CH**_**4**_ **(%)**	55±5.0	50±5.0	61±5.0	62±5.0	61±5.0	55±5.0	51±5.0	66 ±6.0
**CH**_**4**_ **yield (L Kg**^**-1**^**VS)**	163±16	121±12	164±16	293±30	520±50	80±8.0	114±10	516 ±50
**NH**_**4**_^**+**^ **(mg L**^**-1**^**)**	730±37	717±36	847±42	901±45	800±40	1014±50	997±50	878 ±44
**Alkalinity (mg L**^**-1**^**)**	1680±84	1670±83	2470±120	1980±99	2112±6	2650±133	1980±99	2112±106
**Volatile Solid (mg L**^**-1**^**)**	7.40±0.4	9.1±0.5	9.4±0.5	11.3±0.6	11.3±1	8.2±0.4	8.8±0.4	12.7±0.6
**Acetic (mg L**^**-1**^**)**	201.9 ±10	766.9 ±38	22.8±1.44	29.3±1.2	25.7±1	239.5 ±12	268.6 ±13.5	369.4±18
**Propionic (mg L**^**-1**^**)**	21.1±1.0	363.9 ±18	12.2±	n.d	2.8±0.1	621.8 ±31	912.5±46	778.3±39
**Isobutyric (mg L**^**-1**^**)**	n.d	117.4 ±6.0	n.d	n.d	n.d	104.3 ±5.0	170.5 ±8.5	96.2±2.0
**Butyric (mg L**^**-1**^**)**	n.d	91.8±5.0	n.d	n.d	n.d	129.5 ±6.5	88.6±4.4	24.4±1.2

The start-up of reactor RTS was carried out after a 40-day adaptation period to thermophilic conditions (data not shown). Although steady production of biogas was attained during the whole experimental period, results were not satisfactory (Fig 2A). The OLR applied was just 0.33 g VS L^-1^ d^-1^ to prevent VFA accumulation.

Fig 2B shows the methane yields obtained from the codigestion systems. Parameters of the reactor performance are also reported in Table 2. The codigestion process was initiated with a 2.5% addition of cosubstrate, and this value was increased to 5%. The RTS system presented some period of superior performance. Although instabilities associated with high VFA concentration when digesting sludge are inferred from Table 2, the addition of the cosubstrate alleviated the inhibitory state. The codigestion under thermophilic conditions (RTS:F) during the first 30 days showed lower gas yields and higher VFA accumulation, which may be explained by the adaptation needed for the system to degrade complex lipids. This reactor was previously submitted to a 40-day acclimation period to adapt the system to the increase in temperature, therefore the effect associated to VFA build-up is explained by the change in the feeding conditions. Angelidaki and Ahring [27] also reported the need for an adaptation period to attain the degradation of lipids when adding a fatty cosubstrate to the digestion of cattle wastes. Oleate and stearate affected growth by increasing the lag-phase, which implies a shock load of fat can make a biogas reactor inactive for long periods.

The concentration of VFA from the different thermophilic systems was higher when compared to mesophilic reactors, but this is not a characteristic associated with a detrimental impact on performance. Thermophilic reactors present a higher content of VFA during normal operation, as reported by several authors [26, 28, 29]. The addition of a cosubstrate resulted in higher values of propionic acid, but the content in acetic acid was reduced. The accumulation of these acids may be explained by the increase in OLR when the cosubstrate is added. Ammonium values in thermophilic reactors were far from reaching inhibitory levels, so this fact should be discarded as a possible explanation for the poor reactor performance.

The better performance experienced when fat was added might indicate a significant effect of the C/N ratio on the thermophilic performance. Systems codigesting fat presented an increase in biogas production with the increase in cosubstrate addition. Methane yields were 520 L CH_4_ kg^-1^ VS for the mesophilic system and 516 L CH_4_ kg^-1^ VS for the thermophilic system. These results were significantly higher than those obtained from the reactor digesting sludge (163 L CH_4_ kg^-1^ VS for the mesophilic system and 121 L CH_4_ kg^-1^ VS for the thermophilic system) in which great difficulties were encountered. The addition of animal fat not only allowed higher gas production but also increased the stability of the digestion process. Results were in accordance with several studies of sewage sludge codigestion with agro-industrial waste and high lipid content waste. Those studies reported a methane potential between 400 to 500 L CH_4_ kg^-1^ VS for animal fats and around 300 to 900 L CH_4_ kg^-1^ VS for grease trap wastes [7, 23, 30–32]. However, it should be borne in mind that there is always a limit to which addition of wastes can cause a detriment in process performance based on the complex characteristics of the cosubstrate. In the present research, the addition of the cosubstrate resulted in a significant increase in OLR from 0.94 to 1.2 gVS L^-1^ d^-1^ for the mesophilic system and 0.8 to 1.1 gVS L^-1^ d^-1^ for the thermophilic system, which led to higher biogas production, but the main effect was the increase in the stability of the digestion process.

The formation of flocculent aggregates (FW) of fat was observed during thermophilic digestion. Because of the sampling conditions, data reported regarding the performance of RTS:F reactor is affected by the separation of these fatty aggregates in the reactor liquor which remained trapped inside and only were noticeable at the end of the experiment. Data reported in Table 2 for this reactor refer to the characteristics of the effluent which is withdrawn from the sampling port. The presence of the aggregates might be attributed to the high content of LCFAs inside this reactor. Pereira et al. [10] reported that flock structures accumulated lipid material when operating with suspended anaerobic biomass in the presence of high LCFA content. Lipid components can be entrapped by this type of sludge more than by granular sludge. Pereira et al. [11] also observed the accumulation of LCFAs, adsorption onto sludge, and entrapment in flocculent aggregates when palmitic and oleic acids were present in a concentration range of 100 to 900 mg L^-1^.

Table 3 shows the results of LCFA and fat content of digested samples and the fatty aggregate obtained at the end of the experiments upon disassembly of the reactors. A higher concentration of stearic and palmitic acid in the RTS:F digestate and FW aggregate can be observed. No inhibitory effects were observed in the thermophilic reactor during the last 60 days of operation. This could be due to the entrapment of LCFA into FW aggregates, thus reducing the availability of this toxic compound to anaerobic microflora.

**Table 3:** Long chain fatty acids at digested samples.

LCFA (mg L^-1^)	TS	RMS:F	RTS:F	FW
**Caprylic C:8**	20.9±1.0	3.5±0.2	n.d	34.2±1.7
**Capric C:10**	n.d	2.5±0.1	n.d	21.7±1.0
**Lauric C:12**	3.4±0.2	8.5±0.4	n.d	25.9±1.3
**Myristic C:14**	19.1±0.9	60.3±3.0	87.44±4.4	434.1±22
**Palmitic C:16**	223.0±11	536.0±27	663.70±33	3042.7±150
**Stearic C:18**	405.3±20	684.0±34	1952.03±98	7608.9±380
**Fat (%)**	3.2±0.2	3.2±0.2	5.2±0.3	17.7±0.9

### FTIR analysis

FTIR spectroscopy was used to obtain information on the functional groups of organic matter contained in digestates and fatty aggregates, to correlate principal functional groups of fat and sludge, and to assess the hypothesis of inhibition due to fat adsorption.

**Fig 3:**
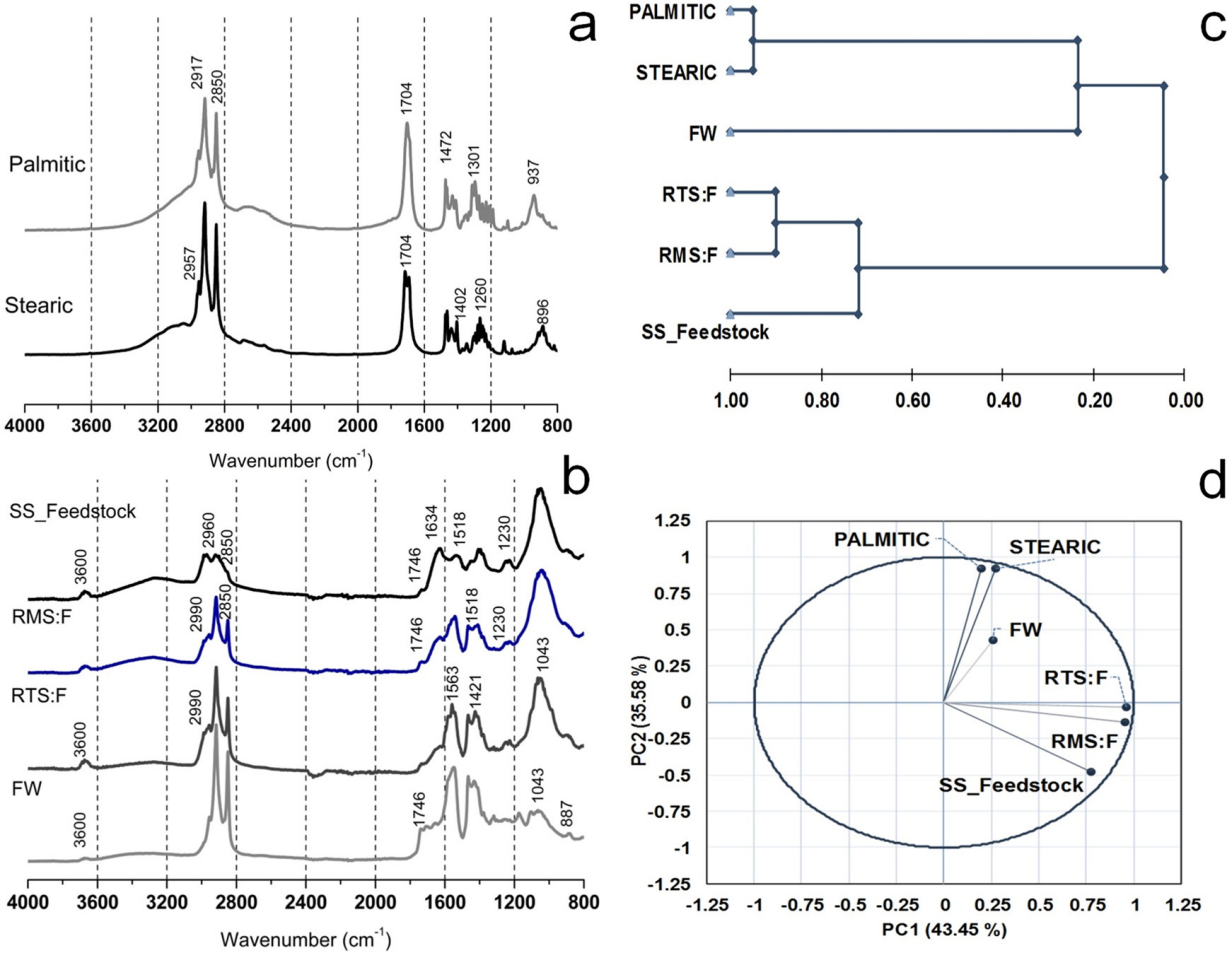
**FTIR spectra of the samples:** (a) commercial LCFAs (Palmitic and Stearic acids) and (b) sludge samples (SS_feedstock, RMS:F, RTS:Fand FW) together with statistical analysis of FTIR spectraby HCA (c) and PCA (d).

Fig 3A and 3B show the FTIR spectra of commercial LCFAs and digested samples and illustrate the associated dominant spectral features. Table 4 presents band assignation.

**Table 4:** FTIR bands assignment for functional groups in digestates and LCFA samples.

Nominal frecuency of bands (cm^-1^)	Assigment^a^
**3600**	O-H stretching of water
**3400**	H-bonded OH groups of alcohols, phenols and organic acids, as well as H-bonded N-H groups
**2971**	Methyl (-CH_3_) asymmetric stretching of lipids
**2917**	Methylene (-CH_2_) asymmetric stretching of lipids
**2850**	Methylene (-CH_2_) symmetric stretching of lipids
**1700–1750**	C = O stretching vibrations of carboxylic groups involved in an ester linkage
**1660–1628**	C = O vibrations of primary amides at sludge
**1548**	C = O vibrations of primary amides
**1450–1410**	CH_2_ scissor deformation vibrations
**1230**	Phospholipids (PO2) asymmetric stretching, protein amide III band (C-H and N-H)
**1070–1048**	–C–O–C of carbohydrates, Si–O–C groups
**888–738**	Scissoring deformation of CH_2_

^a^Assigment was performed according to Guillen and Cabo [33], Tandon et al. [34], Socrates [35], Jouraiphy et al. [36], Francioso et al. [37], Martínez et al. [23], De Oliveira [38] and Hernández-Martínez et al. [39].

Spectra obtained from LCFA samples (Fig 3A) were used to characterize fatty materials. Palmitic and stearic samples showed a high degree of aliphatics with a strong absorption band at around 2800 and 3000 cm^-1^, which is related to C–H stretching. In particular, a band of strong intensity at around 2917 cm^-1^ was observed, which was ascribed to the C–H stretching of aliphatic methylene, and the additional band at 2851 cm^-1^ was assigned to the stretching of aliphatic C–H bonds. The main band identified at 1704 cm^-1^ was ascribed to its carboxylic nature.

Fig 3B shows the spectra obtained from SS feedstock and digestate samples that were taken at the end of semi-continuous digestion experiments. The region ascribed to aliphatic components (2800 to 3000 cm^-1^) is observed in these samples. Digestate samples containing fat as a cosubstrate present a band with higher intensity. The higher intensities registered for these bands obtained from the TS:F and FW samples suggest the adsorption of fat components onto the active sludge, and as a consequence, the degradation of organic material was not completely achieved during the anaerobic treatment. In spite of this, no inhibitory conditions were observed during the operation of the thermophilic reactor, indicating that the formation of the fatty aggregates served to protect the microbial system.

Digestate samples presented two high-intensity bands at around 1560 and 1500–1400 cm^-1^. The first band was ascribed to the presence of proteins in the samples (primary amines) and the second one was ascribed to the adsorption of lipids onto the biomass surface. This peak is also easily discerned in the spectrum from the FW sample. The range below 1500 cm^-1^ is significant for deformation, bending, and ring vibrations. Digested samples and SS feedstock presented a band with an important contribution to the region at around 1185–900 cm^-1^, and these signals were ascribed to polysaccharide components [40].

Statistical analysis of the FTIR spectra allowed the evaluation of the samples based on their FTIR results in order to study the relationship between the RMS:F, RTS:F, and FW samples with the main functional groups of fat (palmitic and stearic acids) and SS_Feedstock. The Pearson’s correlation matrix in Table 5 reports a high correlation between both digestates and SS_Feedstock. In addition, Table 5 shows a significant correlation between FW and RTS:F samples, which is in accordance with the high formation of fatty aggregates found in the thermophilic reactor.

**Table 5:** Correlation matrix (Pearson (n)).

	STEARIC	PALMITIC	SS_feedstock	RMS:F	RTS:F	FW
**STEARIC**	**1**					
**PALMITIC**	**0.950**	**1**				
**SS_Feedstock**	-0.155	-0.218	**1**			
**RMS:F**	0.133	0.075	**0.755**	**1**		
**RTS:F**	0.184	0.106	**0.682**	**0.900**	**1**	
**FW**	0.268	0.201	-0.172	0.114	**0.339**	**1**

Values in bold are different from 0 with a significance level alpha = 0.05

Fig 3C shows the dendrogram of the HCA, while Fig 3D shows the PC plot from the PCA analysis of the FTIR spectra. RMS:F and RTS:F samples are very close and form a group with SS_Feedstock due to the similarity of their FTIR spectra. Likewise, although the distance is slightly greater, samples of palmitic and stearic acids form a group with the FW sample according to the spectra results.

## Conclusions

The codigestion of sewage sludge (SS) with fat (F) was successfully performed under batch and semi-continuous conditions. The SS used in the experiments proved to be difficult to degrade with a high tendency for VFA build-up. The addition of the cosubstrate improved the digestion stability and biogas yield via an increase in the organic loading rate (OLR). However, the thermophilic digestion was characterized by an effluent of poor quality that was associated with high VFA content.

Results from spectroscopic analysis suggested the adsorption of lipid components onto the sludge biomass, which prevented the complete degradation of substrates during the anaerobic treatment. However, the formation of fatty aggregates in the thermophilic reactor avoided the process failure caused by the toxicity of high concentration of LCFAs.

The implications of this work are the feasibility of using discarded butchery fat wastes as a cosubstrate in conventional anaerobic digesters. The addition of the cosubstrate aided in the stable performance of the process under mesophilic and thermophilic conditions. The digestate was characterized by the accumulation of fat, indicating incomplete degradation. This feature would make it unsuitable for agronomic valorization, therefore other alternatives as it is energy recovery from pyrolysis or combustion of the digestate should be explored.
